# Cytokine-induced ‘bystander’ senescence in DDR and immuno-surveillance

**DOI:** 10.18632/oncotarget.1487

**Published:** 2013-10-16

**Authors:** Zdenek Hodny, Sona Hubackova, Jiri Bartek

**Affiliations:** Department of Genome Integrity, Institute of Molecular Genetics, v.v.i., Academy of Sciences of the Czech Republic, Prague, Czech Republic; Department of Genome Integrity, Institute of Molecular Genetics, v.v.i., Academy of Sciences of the Czech Republic, Prague, Czech Republic and Danish Cancer Society Research Center, Copenhagen, Denmark

Cells with damaged and unrepaired genomes represent a potential threat for the organism and therefore their elimination is beneficial. The biological safeguard barriers responsible for elimination of such hazardous cells rely on two principles: intrinsic - achieved through cellular senescence/apoptosis, and extrinsic - performed by the immune system. The concept of immune system-mediated clearance of senescent cells is well established [1], however the exact mechanisms and modes of mutual interplay between cellular senescence and immune surveillance are only emerging.

Research performed in the last two decades shows that cellular senescence as an essentially irreversible block of cell proliferation can be triggered or bypassed via manipulation of expression levels of several dozens of genes indicating the complexity and redundancy of regulatory machineries controlling this antitumor barrier. While the regulatory circuits are not understood in much detail, the unifying feature of cellular senescence is the activation of cell cycle checkpoints that block cell-cycle progression at G1/S or G2/M boundary. Checkpoint activation reflects suprathreshold long-term expression, commonly triggered by persistent DNA damage response (DDR) signaling, of protein inhibitors of cyclin-dependent kinases (CDKs), the key drivers of cell-cycle progression. The multiple pathways leading to the induction of individual (or several) inhibitors of CDKs (CDKi) form the basis for redundancy of mechanisms for induction and maintenance of senescence. The robustness of response, manifested as the possibility to impose senescence even in tumor cells lacking two pivotal senescence mediators p53 and Rb, stems from the fact that the regulatory circuits are interconnected by numerous cross-talks of signaling pathways and several feedback mechanisms [2].

**Figure 1:**
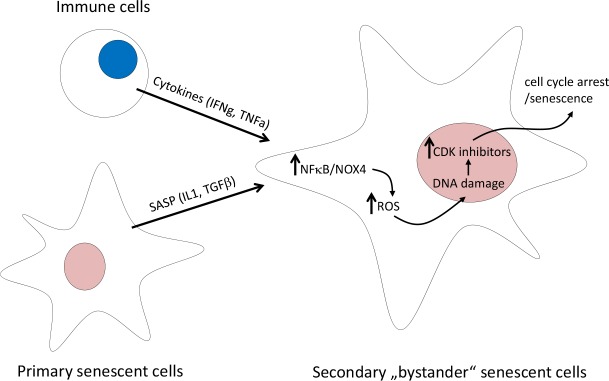
Mechanism of cytokine-induced bystander senescence

Cytokines secreted by senescent cells play here the important role not only in shaping the senescent phenotype by autocrine and paracrine signaling and reinforcing the cell-cycle block by secondary induction of diverse CDKi, but also, as Hubackova *et al*. reported recently [3], by causing genotoxic stress capable of inducing cellular senescence *per se* (Fig. 1). In this study, TGFβ and IL1β, but not IL6, were the two identified cytokine species responsible for secondary/bystander senescence of human diploid fibroblasts. Indeed, exposure of BJ fibroblasts *in vitro* to either of the two cytokines led to development of persistent DDR originating from increased cellular levels of reactive oxygen species (ROS). Production of ROS in cells during inflammation contributes to aging and development of age-related diseases. Nox4 is a member of the NADPH oxidase family known to regulate production of ROS, especially superoxide forms to induce DNA damage and premature senescence. Weyemi *et al*. showed that knock-down of NOX4 decreased RAS-induced DDR [4]. Recently, Kodama *et al*. reported that Ras-induced senescence is mediated via Nox1 and Nox4, and overexpression of both genes is sufficient to induce senescence via activated DDR [5].

Primary senescent cells with persistently activated DDR signaling from unrepaired DNA lesions produce a plethora of cytokines, the senescence-associated secretory phenotype; SASP. IL1beta and TGFbeta induce NADPH oxidase Nox4 in a paracrine manner in normal neighboring cells. Nox4-generated reactive oxygen species (ROS) trigger DDR, whose persistence activates secondary senescence with secondary SASP [3]. In vivo, immune cells (T1 helper lymphocytes) induce senescence in beta-tumor cells by IFNgamma and TNFalpha [6]. A similar mechanism of senescence promotion mediated by IL1beta- and TGFbeta-induced Nox4/DDR is suggested (see text for details).

Even though Hubackova *et al*. did not explore the presence of cytokine-induced secondary bystander senescence *in vivo*, this can be anticipated based on the recent study of Braumuller *et al*. [6]. The cytokines produced during genotoxic stress-induced senescence are among critical regulators of immune system and are innately produced by immune cells. Unexpectedly, Braumuller *et al*. show that T-helper-1 cell cytokines TNFα and IFNγ cooperatively induce senescence in mouse beta-cell tumors, both *in vivo* and *in vitro*, indicating a reciprocal relationship between the immune system and cellular senescence. Importantly, the concerted action of these two T-cell produced cytokines induced senescence in cancer cells indicating the role of immune surveillance in control of tumor cell proliferation by cytokine-induced senescence. Although not assessed in their study, it can be predicted based on previous reports that the observed senescence-inducing effect in response to both IFNγ and TNFα is also triggered by DNA damage. TNFα and IFNγ were also found to increase both intracellular and extracellular ROS production. Importantly, binding of NFκB, a crucial mediator of cytokine effects, on NOX4 promoter was observed [7]. Thus, NFκB activation triggered by upstream cytokine signaling pathways may represent an important upstream trigger of the complex cascade of events promoting senescence. Founded on these two studies, several other combinations of senescence-associated cytokines competent to trigger similar DNA damaging effects and bystander senescence are likely to be revealed in the near future.

It is becoming widely accepted that secretome of senescent cells can also modulate the microenvironment, in both normal or tumor tissues. However, due to the overall complexity and variability of the secreted cytokine species, dependent on specific cell types, (patho)physiological context and senescence-inducing stimulus, it is currently hard to predict all outcomes of senescence-associated cytokine signals on tissue homeostasis. Nevertheless, what can be conceived now, is that the genotoxic effects of several cytokine species produced by senescent cells can spread damage in tissues manifested as secondary (and tertiary) senescence and thus to contribute to aging and pathogenesis of aging-associated diseases. Therefore, the elimination of senescent cells from the organism may provide a rejuvenation effect, as has already been documented in progeroid mice [8].
